# Dynamic coupling between the COVID-19 epidemic timeline and the behavioral response to PAUSE in New York State counties

**DOI:** 10.1371/journal.pone.0255236

**Published:** 2021-08-04

**Authors:** Anca Rǎdulescu, Shelah Ballard, Kaitlyn Gonzalez, Johnathan Linton

**Affiliations:** 1 Department of Mathematics, SUNY New Paltz, New Paltz, NY, United States of America; 2 Department of Biology, SUNY New Paltz, New Paltz, NY, United States of America; 3 Department of Biology, Orange County Community College, Middletown, NY, United States of America; Nanyang Technological University, SINGAPORE

## Abstract

Behavioral epidemiology suggests that there is a tight dynamic coupling between the timeline of an epidemic outbreak, and the social response in the affected population (with a typical course involving physical distancing between individuals, avoidance of large gatherings, wearing masks, etc). We study the bidirectional coupling between the epidemic dynamics of COVID-19 and the population social response in the state of New York, between March 1, 2020 (which marks the first confirmed positive diagnosis in the state), until June 20, 2020. This window captures the first state-wide epidemic wave, which peaked to over 11,000 confirmed cases daily in April (making New York one of the US states most severely affected by this first wave), and subsided by the start of June to a count of consistently under 1,500 confirmed cases per day (suggesting temporary state-wide control of the epidemic). In response to the surge in cases, social distancing measures were gradually introduced over two weeks in March, culminating with the PAUSE directive on March 22nd, which mandated statewide shutdown of all nonessential activity. The mandates were then gradually relaxed in stages throughout summer, based on how epidemic benchmarks were met in various New York regions. In our study, we aim to examine on one hand, whether different counties exhibited different responses to the PAUSE centralized measures depending on their epidemic situation immediately preceding PAUSE. On the other hand, we explore whether these different county-wide responses may have contributed in turn to modulating the counties’ epidemic timelines. We used the public domain to extract county-wise epidemic measures (such as cumulative and daily incidence of COVID-19), and social mobility measures for different modalities (driving, walking, public transit) and to different destinations. Our correlation analyses between the epidemic and the mobility time series found significant correlations between the size of the epidemic and the degree of mobility drop after PAUSE, as well as between the mobility comeback patterns and the epidemic recovery timeline. In line with existing literature on the role of the population behavioral response during an epidemic outbreak, our results support the potential importance of the PAUSE measures to the control of the first epidemic wave in New York State.

## Introduction

Early epidemic development is typically characterized by an initial period with rapidly growing daily incidence, after which the growth rate subsides to a slower curve, reaches a peak, and eventually transitions to a decreasing trend (as the epidemic is dying out). It is common that this first “wave” of the outbreak is followed by others, the timing, size and dynamics of which depend on a variety of epidemic factors (whether the virus confers immunity or not, whether treatment or prevention plans are available), demographic factors (population density and birth rate), control and mitigation factors (presence and efficiency of social distancing measures). The first confirmed infections with SARS-CoV-2 were identified in the US at the end of January 2020. Over the first few months, the outbreak spread dramatically to all US states and territories. The first response to the overwhelming pandemic seemingly attempted to compensate for the clinical unpreparedness (absence of a treatment or prevention plan, notorious limitations in testing, health care resources reaching capacity). Since efficient containment failed, the response consisted of a system of mitigation measures based around social isolation (travel bans, shut down of non-essential businesses, prohibition of large gatherings). The general population was asked to either quarantine or observe social distancing, depending upon suspicion of active symptoms, and upon the gravity of the local situation. In the context of this mitigation process, natural questions have focused around: (1) people’s degree of responsiveness to the centralized directives (including the geographic and social distribution of the response, and the longitudinal patterns) and (2) the effect of the response on the epidemic outcome.

“Social distancing” designates a behavioral response aimed at avoiding potentially infectious contacts, based on available information on an ongoing epidemic. Maintaining physical distance between any two individuals and avoiding mass gatherings are typical social distancing measures; these were recommended in conjunction with the COVID-19 outbreak, together with using facial coverings and maintaining personal hygiene. These measures have been identified historically and predicted by theoretical simulations [1, 2] to be efficient in mitigating an emerging outbreak. Behavioral epidemiology identified behavior of individuals with respect to social distancing as a key factor in predicting trajectories of infectious diseases [3, 4]. For example, studies of the 1918 Spanish influenza proposed that human behavioral response was a key factor behind the temporal changes in transmission rates leading to multiple epidemic waves [5, 6]. Studies of the 2002–2003 SARS epidemic [7] and of the 2009 H1N1 influenza outbreak [8] also concluded that the human behavioral response to incidence of infections could have a significant impact on the longitudinal dynamics and on the magnitude of the epidemic.

The idea that the population behavioral response is a crucial coupling link between health regulations and epidemic dynamics may be viewed as part of a broader psychological and behavioral context. There are general frameworks which aim to contextualize and understand people’s responses to illness (or threat of illness), and their compliance with medical treatments or prevention strategies. The Health Belief Model [9], for example, is based on the fact that each individual’s course of action often depends on their perception of health risk (e.g. severity of symptoms, or susceptibility to illness) versus the benefits related to a health behavior (e.g. curing an existing illness, or diminishing the threat of disease), and the barriers associated to adopting the behavior (e.g., treatment side effects, inconvenience or cost).

These constructs can be easily placed in the context of epidemics, and used to interpret the response to social distancing regulations as likely to be driven by each individual’s assessment of exposure risk, of expected consequences of contracting the virus, and of potential treatment efficiency [10, 11], but also of the impact that following regulations may have on the individuals’ livelihood and quality of life (social, physical and mental wellbeing [11, 12]). Within the latter component, aspects such as the timeline and severity of regulations may play a crucial role in the assessment [10, 13, 14]. This may subsequently affect the person’s affiliation to one health behavior or another (e.g. abiding or refusing to abide by quarantines, masking and other social distancing rules). Both types of response, as well as a continuum between them, have been observed throughout the history of epidemic outbreaks which involved “centralized” measures.

The analogy with the Spanish flu is one most popularly drawn in conjunction with the COVID-19 pandemic [15]. When social distancing measures were introduced in 1918 to stop the deadly influenza outbreak in its tracks, compliance to health mandates was generally high, but pushback existed across the US. Those opposing the mandated measures summoned the effects of the restrictions on the economy, on communities and on individuals’ quality of life. Various demonstrations produced strong enough opposition to force public health officials to roll back orders too early, disrupting a reportedly efficient response to what might have otherwise been a fairly tractable pandemic [16, 17]. These social dynamics around mandated restrictions bears a striking resemblance to those around stay-at-home orders and other guidelines issued in 2020 to mitigate the spread of COVID-19.

The dramatic differences in the timeline and magnitude of the COVID-19 epidemic across different states are quite evident. These differences are likely based on a combination of inherent factors, from timing of the first infection (earlier states were caught unprepared), to population density and intrinsic social dynamics, but also on state-centralized factors (timing of state-wide mandated directives, availability of testing) and on differences in the population’s behavioral response. As some states transcended the first epidemic peak and got a respite in daily infection rates, other states started to experience dramatic surges in numbers. Whether these surges were a refueling of the first infection wave, or distinct, subsequent waves altogether, each brought into discussion the possibility of instating new lockdown measures (or strengthening existing ones), in an attempt to avoid exceeding medical resources and health care capacities (which was the case in New York State in March and April 2020).

New York State had undoubtedly the most dramatic early surge (peaking at over 11K infections per day in mid April, 2020), but was seemingly able to efficiently curb this first wave, with consistently under 1.5K infections per day starting on June 5th, 2020 (and maintained throughout September 2020). It has been hypothesized that this was the effect of a strict and efficient system of government mandated lockdown and social distancing measures (known as PAUSE), in combination with a carefully staggered reopening schedule. In our study we aim to test this hypothesis across New York counties, based on a correlation analysis between the epidemic data on one hand (assessing the outbreak size and dynamics) and the social mobility data on the other hand (assessing the behavioral response to the state mandated measures).

In terms of policy, New York State’s response to the pandemic came in a few stages, both in terms of testing and of mandated social measures. As social dynamics restrictions, among the first to close in the New York State were university campuses and schools [18, 19], followed by restaurants, gyms and other entertainment venues [20, 21]. Along the third week of March 2020, gathering of gradually smaller sizes were banned, all eventually leading to the PAUSE directive that took effects on March 22nd and shut down state-wide all non-essential activity [22, 23]. PAUSE was fully in effect until the start of June 2020, when the process of staggered reopening started, with stages tailored specifically to the situation in each county or region.

By many expert opinions, the COVID-19 epidemic is still unwinding, and may continue to impact health and societal life until it subsides. Even with the rate of vaccinations currently in place in the US, maintaining social distancing is still being recommended by the CDC (as of April 2021), until the efficiency and length of vaccine-induced immunity are better established, and until transmission between vaccinated and unvaccinated individuals is better understood [24]. In many places outside of the US, cases are still surging. This refuels the interest in understanding to which extent social distancing measures are efficient. In our paper, we study the epidemic dynamics in the state of New York, which was one of the US states most severely affected by the first pandemic wave. We analyze these dynamics in conjunction with the state-wide lockdown and social distancing mandated measures, aiming to establish whether data identifies social distancing measures as a potentially crucial player in controlling the first outbreak wave in New York State. The time period considered by our study spans from the start of the epidemic in New York (the date of the first confirmed positive case was March 1st, 2020), until the end of our study on June 20, 2020 (when the statewide number of infections had consistently remained under 1,500 daily for over two weeks, marking the unofficial end of the first epidemic wave).

**Fig 1:**
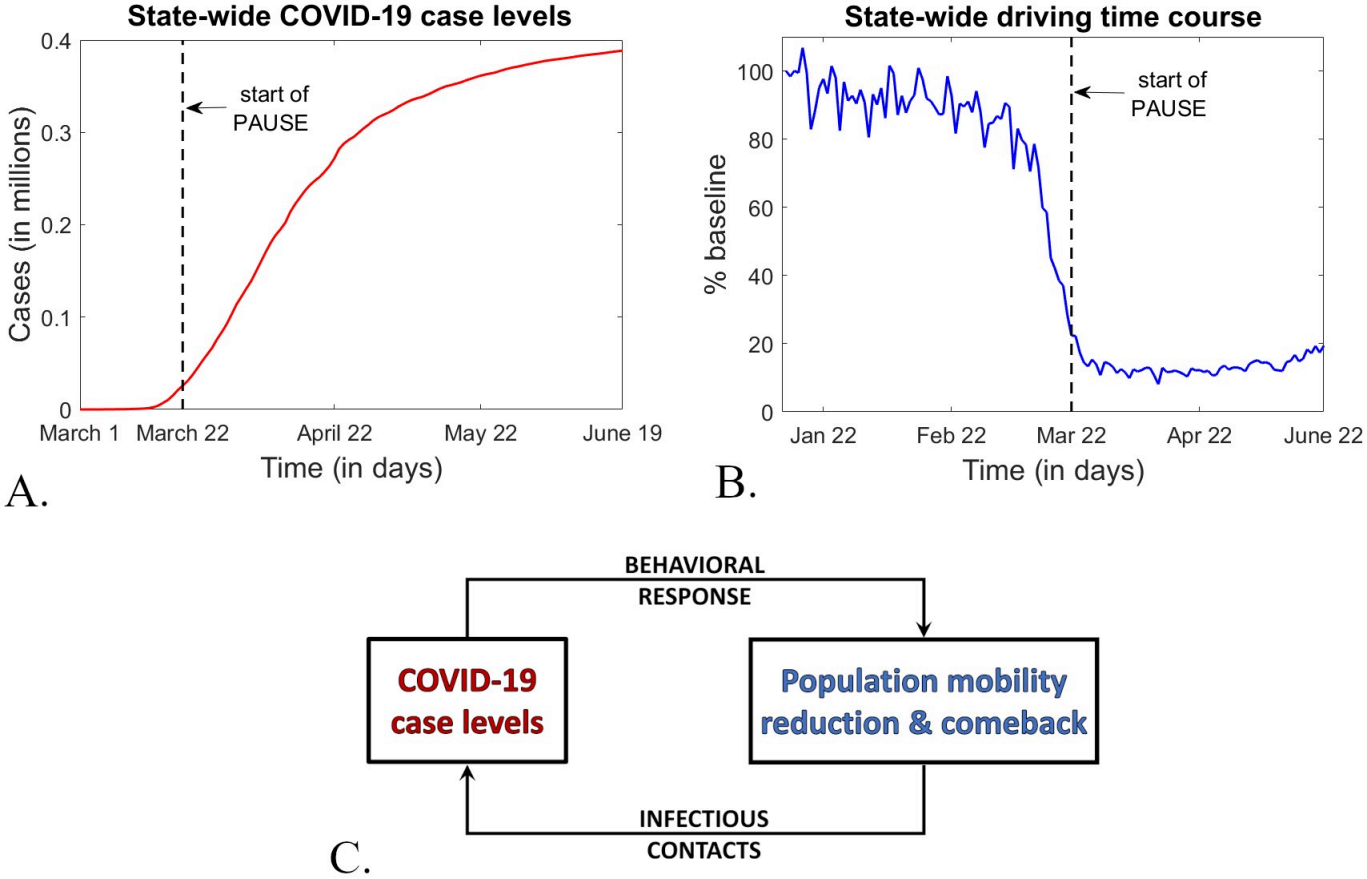
Epidemic and driving time courses in the state of New York, and their potential coupling. **A.** *Total case load, from March 1, 2020 (the day of the first confirmed positive case in the state) until June 20, 2020 (the end of our study)*. **B.** *Driving time course, as reported by Apple (as a % baseline change), from January 13, 2020 (the baseline value) until June 20, 2020 (the end of our study)*. **C.** *Two potential mechanisms that describe bidirectional coupling between epidemic incidence and population mobility during an epidemic outbreak. One aspect of the population behavioral response to increasing case levels is making more careful decisions around leaving the residential space (decisions which may vary even in the presence of general mobility reduction mandates). In turn, reducing mobility affects case levels by reducing the potential infectious contacts within the community*.

We investigate how quickly and to what extent people responded to PAUSE in different counties, and how strictly they observed social distancing measures during lockdown, but also during the reopening stages. We will allow data to inform which social activities had the highest correlations with the existing surge in infections, but also with the subsequent curbing in daily incidence. On one hand, we aim to examine whether different counties exhibited different responses to the PAUSE centralized measures depending on their epidemic situation immediately preceding PAUSE. On the other hand, we explore whether these different county-wide responses may have contributed in turn to modulating the counties’ epidemic timelines (see Fig 1). Our specific goals are to understand (1) to which extent the magnitude of the epidemic immediately preceding PAUSE in each county was correlated with the progression of the social dynamic response to PAUSE measures; (2) to what extent the tightening (during PAUSE) and then relaxation in social mobility (after PAUSE) were correlated with the progression of subsequent epidemic trends.

What makes the assessment of PAUSE efficiency particularly difficult is the absence of control data (we only have real infection data under PAUSE measures, but no estimates of infection counts in absence of social distancing measures). A notorious discussion point centered around the popular misconception that lockdown measures may have been exaggerated (put in perspective of the outbreak decreasing in New York State), when in fact it may have been precisely the presence of those measures that helped control the outbreak.

Establishing with high confidence whether and which social distancing measures were efficient would increase our understanding of the importance of the population response to mitigating the COVID-19 epidemic, and would also help further shape this response. This may apply not only to the ongoing COVID-19 epidemic, but may also contribute to inform our approach to future epidemic emergencies, if necessary. The results of our study support this potential direction of investigation.

The rest of the paper is organized as follows: in the Methods section, we present our data-sets, the characteristics of our time series, and the measures we extracted from these data to assess the size of the epidemic (on one hand), and the efficiency of the state mandated social distancing measures (on the other hand). We also describe the mathematical methods used for data processing and analysis, including the troubleshooting and alternative approaches that we had to design in order to best extract robust patterns from incomplete or imperfect data. We open the Results section by first describing patterns found in each of our data sets considered independently. We first focus on the timeline of the outbreak across counties, since its first New York confirmed infection (March 1st, 2020), until the end of our study (June 20th, 2020). We then investigate the traffic patterns from January 13 until June 20th, 2020 (as provided by Apple); in particular, we identify commonalities and differences between counties. Finally, we describe mobility patterns (as provided by Google), tracking the number of people headed to different targets, as well as their residential time; we aim to understand the psycho-social drives underlying county specific, geography-specific, and week day specific trends. We continue the Results section by showing the outcome of correlational analyses across data-sets, correlations which may reflect the coupling between the epidemic dynamics on one hand, and social dynamics under social distancing measures, on the other. In the Discussion section, we interpret our results, and explore their significance in the context of potential cause-effect relationships between the epidemic timeline, and the efficiency of lockdown.

## Modeling methods

### Data sources and processing

### Epidemic data

**Fig 2:**
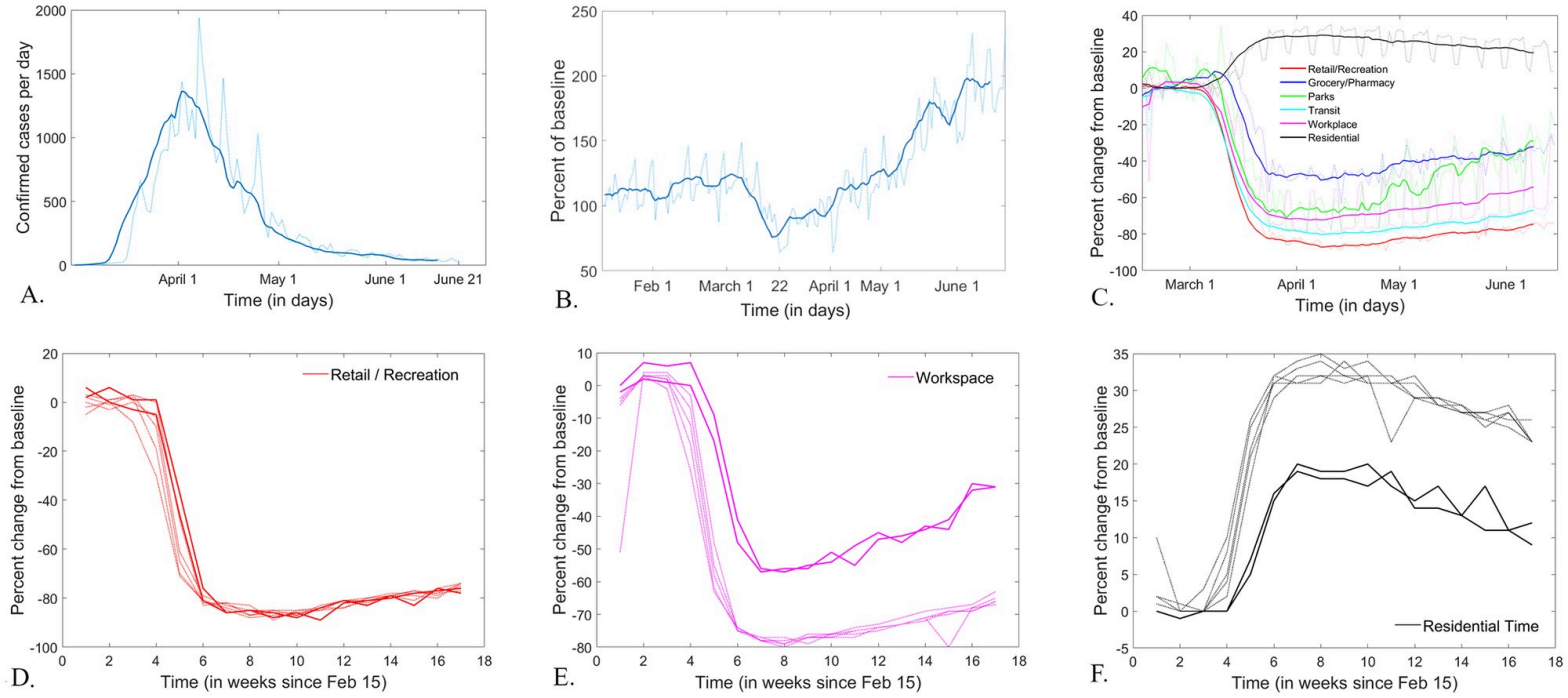
Example of time series and pre-processing, for each data set. **A.** *Example of time series showing the daily incidence of infections, and its windowed smoothing for New York County (i.e., Manhattan). The raw data is represented as a dotted curve; the processed time series obtained by using a seven day average moving window is shown as a solid curve*. **B.** *Example of driving time series and window smoothing for Orange county. The raw data is represented as a dotted curve; the processed time series obtained by using a seven day average moving window is shown as a solid curve*. **C.** *Example of mobility time series and window smoothing for New York County (i.e., Manhattan). The raw time series are represented as dotted curves: in red for Retail; in blue for Groceries; in green for Parks; in cyan for Transit; in pink for Workspace; in black for Residential. The processed time series obtained by using a seven day average moving window are shown as solid curves, with the same color coding*. **D-F.** *Example of weekly mobility time series for Retail (***D**, *in red), Workspace (***E**, *in pink) and Residential mobility (***F**, *in black) in New York County. The Monday-Friday time series are shown as thiner lines, and the weekend time series are shown as thicker lines*.

Time series for cumulative and daily incidence of confirmed infections, as well as for cumulative and daily numbers of COVID-19 tests performed—broken down by New York State county—were accessed from March 1, 2020 (when the infection was first detected in New York) until June 20, 2020 (the end date of our study) from the New York Health Department data repository [25]. Population count and density measures for each county were found in the 2010 New York State census [26]. To track the evolving size of the outbreak, we used, for each county: cumulative incidence (expressed directly as the total number of confirmed cases since March 1, 2020, but also in normalized forms, accounting for the population size, and for the number of tests performed); daily incidence (measured as daily numbers of new confirmed cases, as well as in normalized forms). Since the daily incidence time series showed pronounced weekly oscillations (largely due to corresponding oscillations in reporting), we worked with smoothed time series obtained by using a seven day moving window average, which redistributed these spikes over the short time interval of the moving window, without losing any of the long-term intrinsic epidemic trends (as shown in Fig 2a).

#### Traffic data

Time series describing traffic activity between January 13 and June 20, 2020 were made available in the public domain by Apple [27], based upon their daily numbers of hits for driving / walking / public transit directions requests. The accessible data reports the number of hits originating in each individual county as a percent of the baseline for that county, defined by the county-wide number of hits on January 13, 2020 (but not explicitly provided). While the county-specific baseline makes magnitude comparisons meaningless between different counties, one can still compare the trends in the time series (e.g., one county had a steeper drop than another).

Since the daily time series showed pronounced weekly oscillations, we obtained (as in the case of infection rates) smoothed time series, by using a seven day moving window average, as shown in Fig 2b. One additional problem, which we will discuss later, is the absence of control time series. Since Apple did not make available any data from previous years, it is impossible to interpret the effect of seasonality of annual events (such as increasing trends in traffic with the spring months, or specifically on Memorial weekend).

Records were completely missing for two days (May 11 and 12, 2020) in the Apple traffic reports for all counties. When analyzing these time series, we filled the two missing values by taking the average of the previous and following week readings, before applying the moving window smoothing.

#### Mobility data

Google reports were based in each county on the number of daily searches for directions to five types of destinations (Retail/Recreation, Groceries/Pharmacy, Parks, Transit and Workspace), as well as on the daily amount of time spent at the Residence [28]. These were reported for each county with respect to a set of seven different (week day specific) baselines (not provided in the public domain), computed as the average of the activity on the specific week day for the five week period between January 3 and February 6, 2020. As for the traffic data, the county-specific baselines make magnitude comparisons meaningless, but this still allows the possibility to compare trends between counties. Unfortunately, reports are omitted for days in which the county wide numbers did not meet the privacy threshold. This effect was different in different counties (more pronounced in those with small population size) and for different targets (Parks were most affected, while Retail, Groceries and Workspace were least affected), and its prevalence changed along the timeline. This limited our analysis to the modalities and destinations for which there were sufficient data for an assessment.

An additional difficulty with this data set is the changing baseline, which compromises the longitudinal consistency of the time series. In order to address this problem, we processed the raw time series in two different ways. One was to consider, like in the case of infection rate and traffic time series, a seven window moving average (Fig 2c). However, while this smoothes out the weekly oscillations in the time course and balances some of the baseline discrepancies, it is not a rigorous way to eliminate the dependency on the seven different baselines. Therefore, we also adopted in parallel a different approach, and calculated week day specific time series (e.g., “Sunday weekly series,” or “Monday weekly series”) in which values are sampled at a one week temporal step and reported with respect to the common baseline for the specific day (Fig 2, panels d,e and f). Notice that, for Workspace mobility, the weekend time series appear a lot higher than the Monday-Friday ones. This does not mean that mobility to Work was higher during the weekend, but rather that weekend mobility shows a more shallow drop (since the baseline was already low). Similarly, the weekend Residential time series appear lower than the Monday-Friday ones, reflecting the higher weekend baseline (the typically higher number of residential time during weekends).

### Correlation analysis

In order to measure the strength of the linear association over all New York State counties between two variables (an epidemic measure X and a traffic/mobility measure Y), we used a nonparametric test, the Spearman rank-order correlation (the Pearson correlation coefficient between rank variables):

$$\begin{array}{c} R=\frac{\text{cov}(rk_{X},rk_{Y})}{\sigma_{rk_{X}}\sigma_{rk_{Y}}} \end{array}$$

where *rk*_*X*_ and *rk*_*Y*_ are the rank variables corresponding to *X* and *Y*.

Spearman’s rank-order correlation assesses monotonic relationships (whether linear or not), with a positive value 0 < *R* ≤ 1 when X and Y tend to simultaneously increase, and a negative value −1 ≤ *R* < 0 if Y tends to increase when X decreases. The Spearman correlation increases in magnitude as X and Y become closer to being perfectly monotone functions of each other. We used the Spearman correlation, because it is less sensitive than the Pearson correlation to strong outliers in the tails of both samples (since the outlier is limited to the value of its rank). We report only statistically significant correlations with a significance threshold of *p* < 0.05.

## Results

### Infection trends

**Fig 3:**
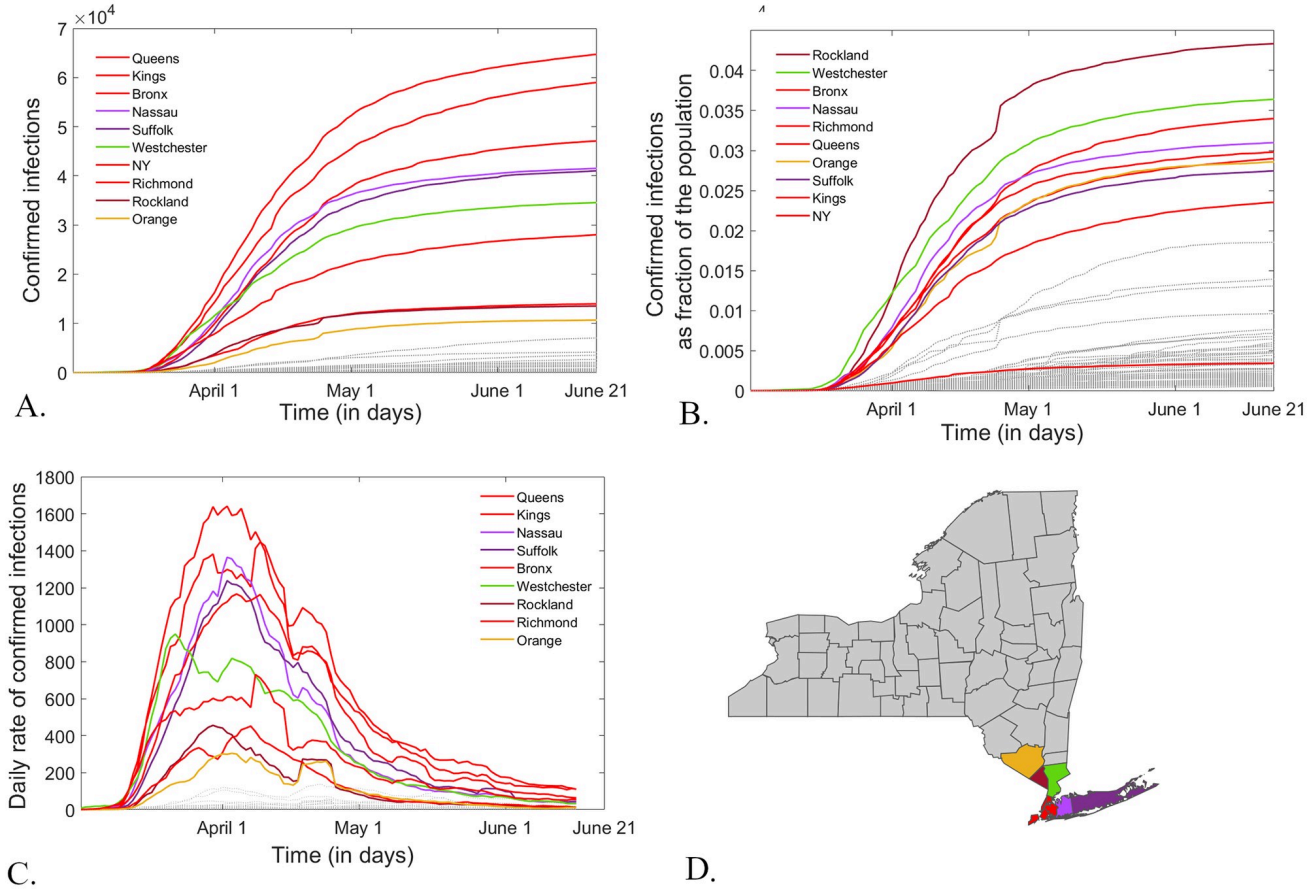
Infection time course in New York counties from March 1 until June 20, 2020. **A.** *Cumulative incidence of COVID-19 infections;* **B.** *Cumulative incidence normalized by the county population size (reported as a percent). The counties with the highest cumulative incidence by June 21 are emphasized as solid curves, and labeled top to bottom in the legend. The rest of the counties, with lower incidence, are shown as faded dotted lines, for comparison*. **C.** *Smoothed time series for daily incidence of COVID-19 in New York counties form May 1 until June 20, 2020. The counties with the highest daily incidence by June 20 are labeled and emphasized as solid curves. The rest of the counties, with lower daily incidence, are shown as faded dotted lines, for comparison*. **D.** *Locations of the high infection counties from panels A, B and C, shown on the New York state map with the same color coding*.

In response to the dramatic increase in COVID-19 incidence in early March 2020, lockdown measures were implemented and maintained through the peak and subsequent decay of the first epidemic wave (March through May, 2020). From the start of June 2020, as the New York State has started its staggered reopening, and up to the end of this study (June 20, 2020), the state-wide cumulative incidence of COVID-19 infections shows an almost asymptotic trend, with steadily decreasing daily incidence (which was in fact one of the reopening benchmarks). While all counties with significant infection show the same slowing down growth to “capacity” in cumulative incidence, Fig 3a illustrates the wide variability in the sheer size among counties. New York City counties lead the first tier, followed within the same order of magnitude by suburban counties (Nassau, Suffolk, Westchester, Rockland, Orange). When normalizing the cumulative incidence by the population size in each county, as reported on the 2010 census (Fig 3b), the order changes slightly (with Rockland and Westchester on top, and New York County less significantly affected, when accounting for its huge population size), but the primary infection hubs identified by this measure are the same as for the raw case counts (with Spearman rank correlation over all counties at the start of PAUSE between the cumulative incidence and the percent cumulative incidence: *σ* = 0.79, *p* < 10^−13^).

A helpful representation of the epidemic dynamics is provided by the daily incidence time series, shown in Fig 3c (recall that these are seven day moving average smoothed time series). These better illustrate the rise and fall of the first epidemic wave in New York, and strongly support the possibility that the outbreak had been at least temporarily controlled. We are interested to investigate whether and how PAUSE may have contributed to this control. Understanding the strategies that successfully worked to curb a daily rate higher than 11K in April 2020 to a daily rate of less than 1K in June 2020 are crucial to controlling the resurgence of infection during the ongoing COVID-19 epidemic, but also for future reference in relation to management of other potential outbreaks.

### Traffic trends

**Fig 4:**
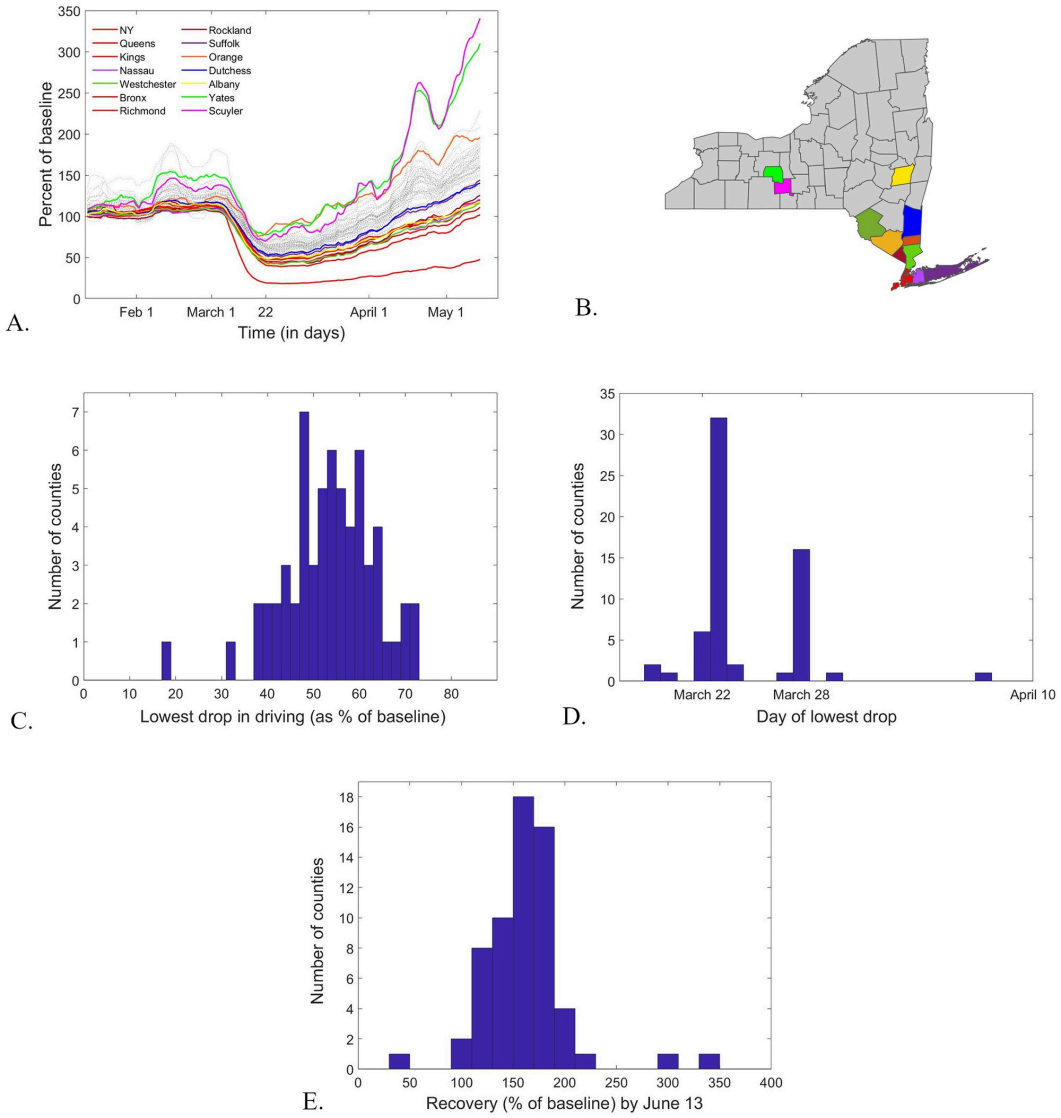
Description of driving trends (as per the Apple data set) in New York counties. **A.** *Time course (of numbers of hits to driving destinations per day with respect to baseline). Each curve shows the timeline for one county: the counties with high infection rates are color coded and shown in the legend; the others are shown as dotted grey lines*. **B.** *The location of each county shown in color in panel A is represented in the same color on the NYS map*. **C.** *Histogram of the lowest driving levels, as fraction of baseline*. **D.** *Histogram of the day in the time course when the lowest driving level (shown in C) occurred for each county*. **E.** *Histogram of the comeback driving level for each county, as of the last day computed by our smooth window averaging (June 13)*.

The Apple driving time courses are highly correlated across New York counties (mean pairwise Searman coefficient $\bar{\rho }=0.9145$ and mean significance *p* = 7.17 × 10^−7^). In broad strokes, they all show a sudden drop from baseline, initiated around March 4th, 2020 (a few days after the first infections were confirmed in New York State), reaching an inflection point around March 12th (the day of the first government-mandated state-wide closures), dropping to a minimum value around March 22nd, 2020 (the day PAUSE was instated), then slowly recovering towards the baseline (or even to values higher than the baseline, in some cases), at various upward rates (depending on the county). To illustrate these common trends, Fig 4 shows simultaneously the smoothed driving time courses for all 61 counties in New York State. For each of these smoothed time series, the lowest traffic level was computed (as a % of the original baseline). Fig 4c shows the histogram of these lowest values, and Fig 4d shows the histogram of the times when the deepest drop was achieved along the smoothed time course. Notice that all the lowest values are reached within a nine day window after March 22 (the outliers are counties which also had close to minimum values within this window). Fig 4e shows the histogram of the % values of the baseline to which traffic had recovered by June 13 (the last data-point we computed from the smoothed time series).

Broadly speaking, all NYS counties show a highly coordinated drop in driving over an approximately two week long time window in March 2020, as an effect of closing many places of business (and subsequently the traffic to and from them). The drop appears to have been initiated prior to the PAUSE directive, and enhanced by the psychological and citizenship response to the increase in epidemic awareness and to the state directed measures.

However, there are also sizable differences between the traffic timelines in each county, in particular in their comeback path from this dramatic drop. While some counties (in particular those with highest COVID-19 incidence, among which the New York City counties) showed a slower upturn rate, others had already (by the end of our study on June 20th, 2020) regained or exceeded the % baseline values (set on January 13, 2020). This is not surprising, since the seasonal level of functional traffic for May/June is expected to be higher than in January during a typical year).

**Fig 5:**
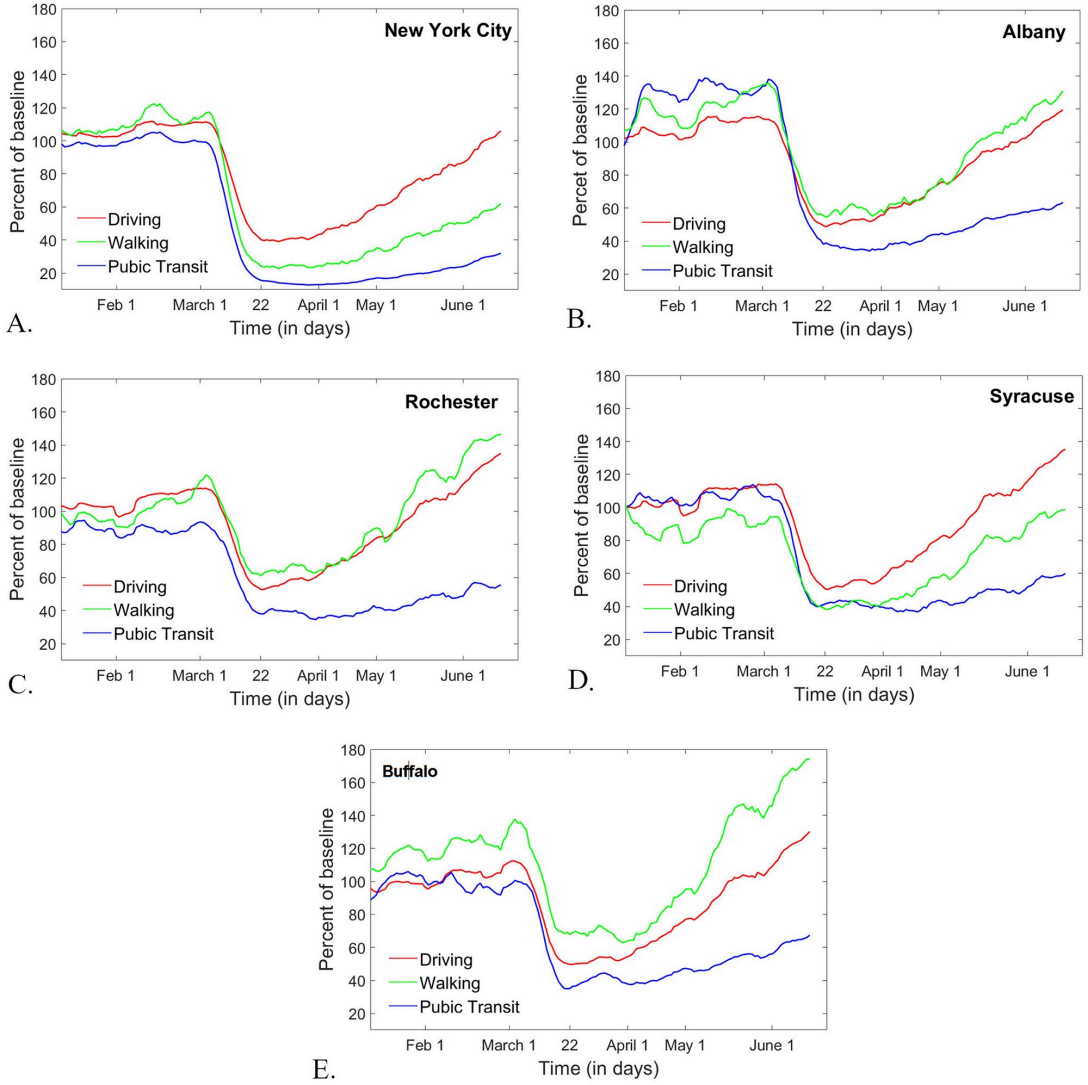
Trends in all traffic modalities (driving, walking and public transport) as per the Apple data set, for five metropolitan areas in New York State. **A.** *New York City;* **B.** *Albany;* **C.** *Rochester;* **D.** *Syracuse;* **E.** *Buffalo. Driving patterns are shown as a red curve, walking in green and public transport in blue*.

The differences in the social dynamic response to the epidemic between different New York regions is also reflected in different traffic patterns for the five metropolitan areas where information was provided on all three modalities (driving, walking and public transport). Fig 5 illustrates how different modalities recovered at different rates for the same city, and how these signatures differ between the five cities. Notice that the reduction and comeback in driving shows similar patterns in all five cases (with the lowest point around the initiation of PAUSE and a comeback to 100% or slightly above baseline by June 20). The public transit suffered the most pronounced drop in all of the five cases, but the level of this drop and the subsequent upturn slope still differ between counties (with New York City showing, unsurprisingly, the most pronounced drop from a very high baseline of public transit, and also showing the slowest comeback (to less than 40% baseline by June 20). The most pronounced differences across these five areas are in walking patterns. Indeed, waking in the New York City shows a very slow comeback to baseline (slower than that of driving), as opposed to Rochester or Buffalo, where walking quickly recovered to values much higher than the baseline (which, as mentioned before, would be expected in absence of the epidemic, due to the seasonal increase with respect to the January-based baseline). This is not surprising, since the amount of walking was the most likely to be influenced by factors like population density, geographic position (proximity to nature, and other large spaces which permit social distancing while walking). Unfortunately, a more in depth, county-wise analysis of walking patterns was not possible, since data was only provided for these five metropolitan areas.

### Location specific mobility trends

Similarly with the traffic timeline, mobility to the five location categories (Retail, Groceries, Parks, Transit and Workspace) was highly synchronized across New York counties, and so was the time duration spent at the residence.

**Fig 6:**
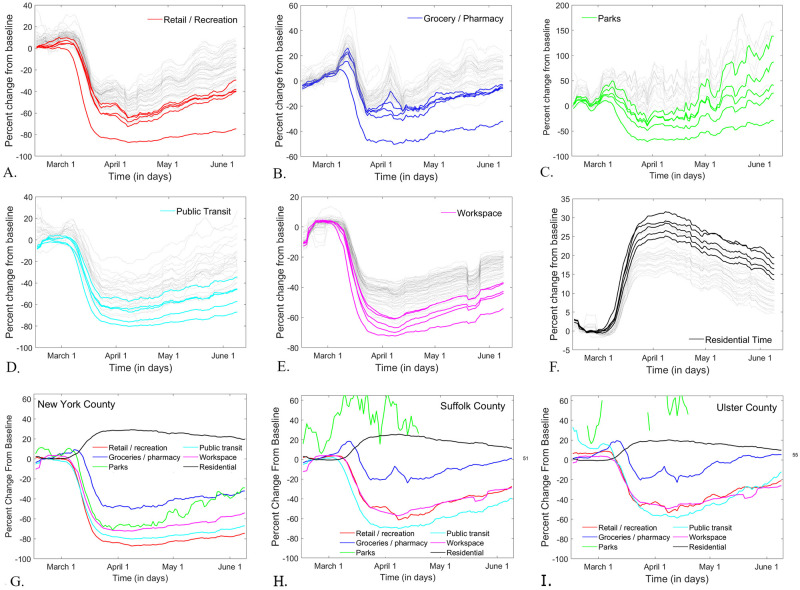
Seven day moving window average time series *describing mobility (as reported by Google) to* **A.** *Retail and Recreation;* **B.** *Grocery and Pharmacy;* **C.** *Parks;* **D.** *Public Transit;* **E.** *Workspace;* **F.** *Residential time. New York City counties are shown in thick solid lines; all other counties are shown in grey dotted lines*. **G-I.** *Seven day moving window average time series for mobility to all destinations for New York County (***G***); Suffolk County (***H***); Ulster County (***I***)*.

**Fig 7:**
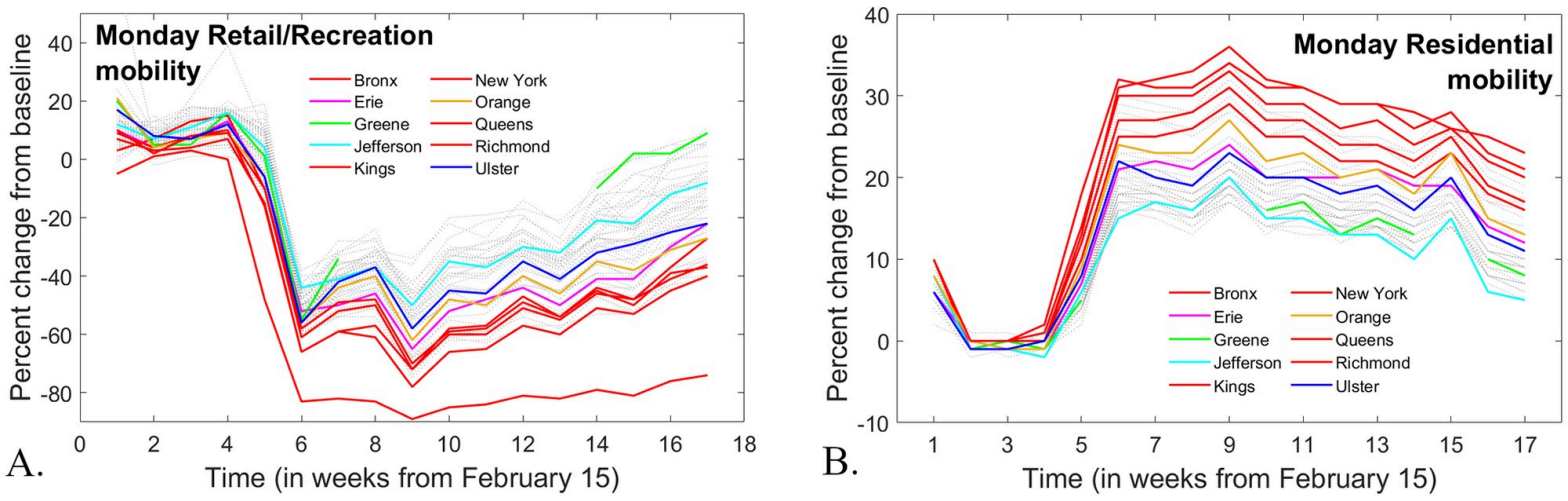
*Weekly Monday mobility trends for all counties, as reported by Google for* A. *Retail and Recreation and* B. *Residential time*.

Fig 6 illustrates simultaneously for all counties the mobility trends to all destinations, as well as the residential time. Each panel represents a destination, and each curve corresponds to the smoothed timeline in a different county. New York City counties (emphasized as solid lines in each panel) were in each case among the counties with the most pronounced effect: deep drop in mobility and high rise in residential time at PAUSE, followed by slow return towards the original baseline afterwards. Recall that New York City counties were also the ones to exhibit the most pronounced cumulative and daily incidence at PAUSE. The same trends were identified when using the weekly representation for mobility to destination and for residential times, as shown in Fig 7. In the next sections, we will investigate whether this was just an isolated, unique phenomenon, specific to New York City, or whether broader correlations exist between mobility and infection, across all counties. In order to avoid placing weight on the New York City counties (which are are the high end of the spectrum in both mobility and infection measures), we used nonparametric, rank correlations.

Another broad and hardly surprising observation is that the trips to retail, work and transit dropped significantly lower than those to groceries and parks. Parks and the outdoors varied widely between counties; as we mentioned before, this is likely based on the geographic position and access to nature (large outdoor spaces where social distance can be observed). These observations are supported by Fig 6g–6i, which compares mobility patterns for all six compartments between three example counties, one in each infection cluster from Fig 3: New York County, Suffolk County and Ulster County. In New York County, for example, mobility to Parks decreased to a comparable extent to mobility to Retail, Transit and Workspace destinations, after which it started a very slow upturn, and remained below the January baseline, even by June 20, 2020. In contrast, mobility to Parks in Suffolk and Ulster Counties shows a more rapidly rising trend from baseline, seemingly unaffected by the onset of PAUSE.

Information on mobility to Parks would be of particular interest, in the context of using the data to assess infection risk during outdoor recreational and other activities. Unfortunately, the Parks time series have many gaps in reporting, which are augmented by the windowed smoothing. Further processing the data to account for these gaps would render any assessment of intrinsic trends unreliable.

### Correlations between epidemic size at onset of PAUSE and lowest traffic/mobility levels after onset of PAUSE

One direction of inquiry is to use these data to study the population behavioral response to the COVID-19 epidemic in New York State. While a causal relationship is difficult to establish, correlations between the size of the epidemic in different counties and the degree of their lockdown may indicate the effect that the perception of risk may have had on the population response to the mandated measures. To explore this, we used measures of the outbreak size described in the Methods section. At the time PAUSE was initiated (March 22, 2020), we computed, for each county: (1) cumulative incidence of COVID-19 infections (*CI*, total number of cases), (2) percent cumulative incidence (%*CI*, total number of cases reported as a population percent); (3) cumulative incidence per tests (*CI*/*T*, total number of cases *CI* divided by the total number of tests performed *T*); daily incidence (*DI*); (4) percent daily incidence (%*DI*); (5) daily incidence per daily tests (*DI*/*DT*). We computed *DI* and *DI*/*DT* for the original time series, as well as for their seven-day window smoothed versions.

Over the first few weeks corresponding to the emergence of the COVID-19 epidemic in New York state, testing was notoriously limited. Reporting incidence as a fraction of the number of tests performed may seem an adequate approach to this small sample issue. This would apply if testing were performed on a random sample of the general population, implying proportionality between incidence and the number of tests. However, testing availability was not independent of the epidemic, and likely scaled up in response to the local number of reported COVID-19 cases, thus correlating with incidence. In this context, we decided to report our main results without this normalization, and include results for *CI*/*CT* and for *DI*/*DT* separately in Appendix A in S1 Appendix, for comparison.

As measures of the PAUSE efficiency, we considered the minimum level of traffic/mobility. This was defined as a fraction of the original baseline, (so that a lower value can be interpreted as corresponding to a more extensive shutdown), and was shown in the Methods section to occur promptly after PAUSE, and be highly synchronized in time across counties, modalities and venues. We set up to compute this minimum value both for the smoothed traffic time series (Apple data), and for the smoothed mobility time series (Google data). Because of the missing data in some of the mobility timelines (primarily due to Google privacy thresholds), we could only sustainably calculate the lowest point for the Retail, Groceries and Workspace time series. While the most variability between counties appears to occur in travel to outdoor Parks, that is also the data which had the largest gaps, making a consistent analysis impossible.

**Table 1:** Spearman rank correlations between our measures of epidemic size and our measures of social mobility in New York counties at the time of PAUSE. The epidemic measures (computed on the day PAUSE started) are: cumulative incidence for each county (*CI*, columns 1 and 2); percent cumulative incidence (%*CI*, columns 3 and 4); daily incidence (*DI*, columns 5 and 6); percent daily incidence (%*DI*, columns 7 and 8). The first row shows the correlations of these epidemic measures with the lowest traffic level (which occurred briefly after the start of PAUSE), as a fraction of the original traffic baseline. The other three rows show the corresponding correlations with the lowest mobility to Retail, Grocery and Workspace (as a fraction of the baseline). The corresponding significance values are shown as separate columns. The correlations with daily incidence *DI* and %*DI* were based on values from the window smoothed time series; the corresponding correlations based on raw time series were very similar.

	*CI*		%*CI*		*DI*		%*DI*	
	Corr coef	p-value	Corr coef	p-value	Corr coef	p-value	Corr coef	p-value
**Traffic min**	-0.64	<10^−7^	-0.63	<10^−7^	-0.67	<10^−8^	-0.63	<10^−7^
**Retail min**	-0.69	<10^−9^	-0.64	<10^−7^	-0.67	<10^−8^	-0.63	<10^−7^
**Grocery min**	-0.47	<10^−5^	-0.64	<10^−7^	-0.47	<10^−3^	-0.45	<10^−3^
**Work min**	-0.81	<10^−14^	-0.75	<10^−11^	-0.83	<10^−16^	-0.72	−10^−10^

**Table 2:** Spearman correlations between measures of epidemic size and weekly measures of social mobility in New York counties at the time of PAUSE. The epidemic measures are: cumulative incidence for each county (*CI*, columns 1 an 2); percent cumulative incidence (%*CI*, columns 3 and 4); daily incidence (*DI*, columns 5 and 6); percent daily incidence (%*DI*, columns 7 and 8). The lowest mobility levels to Retail, Grocery and Workspace were computed separately for each day of the week; the table shows the results for correlations with Tuesday and Sunday time series. The correlations with daily incidence *DI* and %*DI* were based on values from the window smoothed time series; the corresponding correlations based on raw time series were very similar.

	**Tuesday**							
	*CI*		%*CI*		*DI*		%*DI*	
	Corr coef	p-value	Corr coef	p-value	Corr coef	p-value	Corr coef	p-value
**Retail min**	-0.71	<10^−9^	-0.68	<10^−8^	-0.63	<10^−7^	-0.62	<10^−7^
**Grocery min**	-0.46	<10^−3^	-0.48	<10^−4^	-0.49	<10^−4^	-0.43	<10^−3^
**Work min**	-0.82	<10^−15^	-0.77	<10^−12^	-0.73	<10^−10^	-0.75	<10^−11^
	**Sunday**							
	*CI*		%*CI*		*DI*		%*DI*	
	Corr coef	p-value	Corr coef	p-value	Corr coef	p-value	Corr coef	p-value
**Retail min**	-0.53	<10^−4^	-0.45	<10^−3^	-0.45	<10^−3^	-0.45	<10^−3^
**Grocery min**	-0.25	0.04	–	–	–	–	–	–
**Work min**	-0.71	<10^−10^	-0.68	<10^−8^	-0.60	<10^−6^	-0.67	<10^−8^

All measures of epidemic size correlated negatively with the lowest traffic and mobility levels; in other words, the higher the infection (be it measured in incidence or percent incidence, as total case load or as daily rates), the lower the traffic/mobility minimum (i.e., the more severe the traffic/mobility drop in activity). This proved to be true for both driving trends (shown in the first row of Table 1), and for mobility to Retail, Groceries and Work (shown in rows 2–4 of Table 1).

As we had mentioned in the Methods section, the Google mobility data is best used and interpreted separately for each week day (since different week days have different baseline values, hence it is problematic to mix, average or compare the baseline percent changes with each other). Therefore, in order to increase the confidence of our correlations, we also did the statistical analysis using week day specific time series.

Table 2 illustrates the correlation values for the Tuesday and Sunday mobility data. The other week days were similar, and are not shown. Notice that Retail and Work correlated most strongly with the epidemic measures. Traffic (a less reliable measure in terms of destination and exposure) showed weaker correlations, and travel to Groceries showed the weakest correlations. Also, the correlations were stronger for the “working” week days than for the weekend days.

Altogether, this first correlation test shows that the degree to which social mobility was restricted was more pronounced in counties in which higher infection and higher infection rates were reported at PAUSE. The correlation was stronger for destinations which did not represent daily necessities, and less pronounced for destination which were either essential (food, medicine) or safer (outdoors). These ideas will be revisited in the Discussion section.

### Correlation between lowest traffic/mobility levels and epidemic recovery after onset of PAUSE

A second important direction of inquiry is to study the potential coupling between the size of the population response to PAUSE and the COVID-19 epidemic trends following the PAUSE shutdown. Along this line, we computed correlations between the lowest drop in traffic/mobility after the start of PAUSE, and measures of the subsequent epidemic progression. While a causal relationship cannot be directly inferred simply from significant correlations, finding a significant coupling between the reduction in population mobility and a reduction in epidemic size following it may suggest that causality is possible, and guide further efforts aimed at investigating this relationship.

The PAUSE directive was introduced with the aim of controlling the spread of COVID-19 within communities, and “flattening the curve.” It was originally suggested that its first effects would be visible within 14 days (the maximum observed incubation period). Looking back, it is important to understand whether PAUSE was effective, and if yes, how fast.

Pursuing this direction of study, we investigated whether deeper traffic and mobility drops in response to PAUSE correlated with better epidemic outcomes, as evaluated from the progression of the epidemic between the start of PAUSE (March 22, 2020) until the end of the study (June 20, 2020). We also investigated whether the data suggest a different estimate of how long after the initiation of PAUSE this correlation started to take effect.

**Table 3:** Spearman correlations between lowest traffic/mobility levels in New York counties shorty following PAUSE, and epidemic control by the end of our study. As measures of epidemic control, we used the percent change in cumulative incidence %Δ*CI* and the change in smoothed daily % incidence Δ%*DI*, between the start of PAUSE and the end of our study. These were correlated with the lowest traffic level, as fraction of traffic baseline (top row), and with the lowest mobility levels to Retail, Grocery and Workspace (remaining three rows). The corresponding significance values are shown as separate columns.

	%Δ*CI* 90 days after start of PAUSE		Δ%*DI* 83 days after start of PAUSE	
	Corr coef	p-value	Corr coef	p-value
**Traffic min**	0.43	<10^−3^	0.58	<10^−6^
**Retail min**	0.47	<10^−3^	0.53	<10^−7^
**Grocery min**	0.42	<10^−3^	0.39	0.001
**Work min**	0.38	0.002	0.61	<10^−6^

**Table 4:** Spearman correlations between lowest weekly mobility levels in New York counties shorty following PAUSE, and epidemic control by June 20, 2020. As measures of epidemic control, we used %Δ*CI* and Δ%*DI*, between the start of PAUSE and the end of our study. The lowest mobility levels to Retail, Grocery and Workspace were computed separately for each day of the week; the table shows the results for correlations with Tuesday and Sunday time series.

	**Tuesday**			
	%Δ*CI* 90 days after start of PAUSE		Δ%*DI* 83 days after start of PAUSE	
	Corr coef	p-value	Corr coef	p-value
**Retail min**	0.65	<10^−8^	0.48	<10^−4^
**Grocery min**	0.51	<10^−4^	0.41	<10^−3^
**Work min**	0.60	<10^−6^	0.48	<10^−4^
	**Sunday**			
	%Δ*CI* 90 days after start of PAUSE		Δ%*DI* 83 days after start of PAUSE	
	Corr coef	p-value	Corr coef	p-value
**Retail min**	0.41	<10^−3^	–	–
**Grocery min**	–	–	–	–
**Work min**	0.52	<10^−4^	0.50	<10^−4^

To accomplish this we used, as before, the value for each county of the lowest traffic/mobility level as a measure of the population response to PAUSE. We then calculated measures of epidemic “recovery” within the 90 days between the start of PAUSE and the end of our study. Keeping in mind that the traffic minima occurred within the first nine days after the onset of PAUSE, we first estimated, for each day 10 ≤ *k* ≤ 90 following the start of PAUSE, the percent change in cumulative incidence, between its value at the start of PAUSE and its value on the current day *k*:

$$\begin{array}{c} \%\Delta CI\left(k\right)=\frac{100\cdot [CI(k)-CI(1)]}{CI(1)+1} \end{array}$$

(the +1 was included in the denominator in order to avoid obtaining an undefined quantity for counties which had zero cases at PAUSE). We also computed the change in percent daily incidence between its current value on day *k* ≥ 10 and its value at the start of PAUSE: Δ%*DI*(*k*) = %*DI*(*k*) − %*DI*(1). We computed this for the original time series, and for the seven day moving window averages (only results for the latter are shown, the corresponding results for original time series are very similar).

We first focused on understanding how well the population response to PAUSE predicted epidemic recovery by June 20th. We calculated the Spearman correlations between the lowest traffic/mobility measures, and the final values of the epidemic recovery (for %Δ*CI*, the last calculated value was for June 20th, i.e., *k* = 90; for Δ%*DI*, which uses the smoothed *DI* time series, the last day corresponds to June 13th, i.e., *k* = 83). The corresponding correlation and significance values are shown in Tables 3 and 4. The results show that the epidemic outcome in each county by June 20 correlated significantly with the reduction in traffic/mobility at PAUSE: a lower minimum in traffic/mobility can be associated with a milder percent raise in cumulative cases since PAUSE, and with a bigger reduction (i.e., more negative change) in percent daily incidence since PAUSE. Out of all correlations, the weakest among traffic/mobility measures were those with mobility to Groceries.

**Fig 8:**
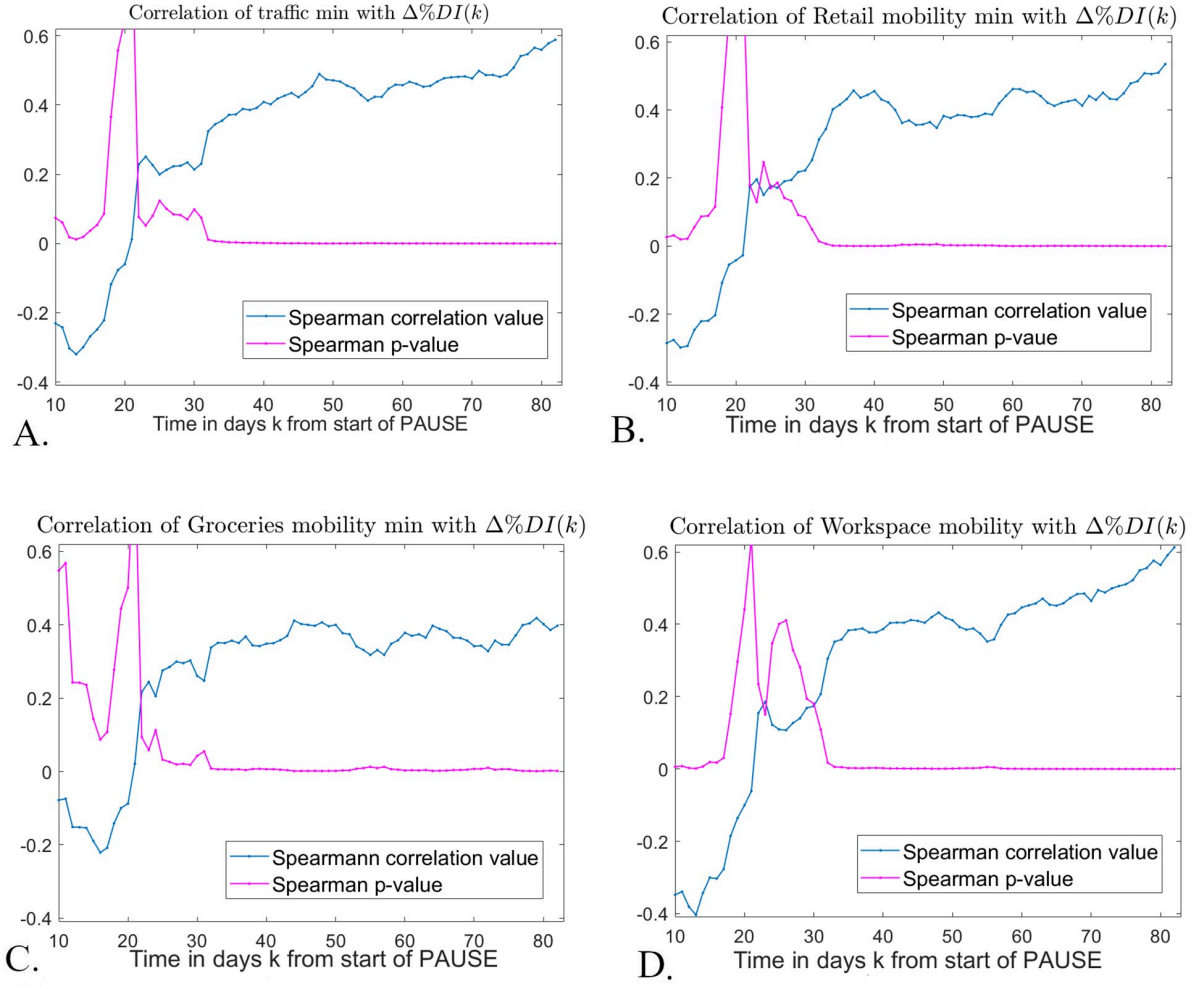
Spearman correlations between traffic/mobility lowest levels after PAUSE, and epidemic recovery before June 20, 2020. *We start our assessment of traffic/mobility comeback at the 10th day after the start of PAUSE, so as to allow for the smoothed traffic/mobility time series to achieved their lowest levels (recall that these occurred within a nine day window from the start of PAUSE, when considering smoothed traffic time series). For each day* *k* ≥ 10 *after the start of PAUSE (represented along the horizontal axis) we computed correlations between the traffic/mobility lowest level and the epidemic recovery measure* Δ%*DI*. *The values of* Δ%*DI were computed from the smoothed epidemic time series, for* 10 ≤ *k* ≤ 83 *days. In each panel, the day* 10 ≤ *k* ≤ 83 *is represented on the horizontal axis; the corresponding correlation and significance values are shown as blue and respectively purple curves. Each panel illustrates the results for a different traffic /mobility data set:* **A.** *driving, as percent of baseline;* **B.** *mobility to Retail;* **C.** *mobility to Grocery;* **D.** *mobility to Workspace, as percents of the reference day*.

Once established that there is an association between the social response to PAUSE (at its lowest point) and the epidemic outcome on June 20, our next question focused on when (at which point between the start of PAUSE and June 20) this coupling became significant. Fig 8 shows the Spearman correlation values (and corresponding significance) between the traffic/mobility lowest drop following initiation of PAUSE and the change in percent daily incidence Δ%*DI*(*k*), for all *k* ≥ 10.

All representations in Fig 8 are phenomenologically similar. For a window of 2–4 days (the exact length depending on the traffic/mobility modality), the lowest level in traffic/mobility is negatively correlated with Δ%*DI*(*k*). These correlations are generally weak, with strength and significance also depending on the modality (with the strongest negative correlations for mobility to Workspace, and the weakest for mobility to Groceries). This is followed by a transient window with no significant correlations between the two measures of interest. Around *k* = 30 days after onset of PAUSE, the correlations finally stabilize to positive values, which remain consistently significant until the end of the observed time interval (*k* = 83). This pattern is not a surprise, since we showed that lower traffic/mobility minima corresponded to higher epidemic measures at the start of PAUSE, and one would not expect this negative correlation to immediately vanish. Instead, the negative correlation values slowly become weaker and eventually disappear, to be replaced around 30 days from the onset of PAUSE by positive correlations, likely due to a different underlying mechanism. More precisely, after about four weeks from the start of PAUSE, epidemic recovery starts to show significant positive correlations with the extent of the mobility drop from PAUSE, in the sense that more severe drops in mobility were associated with more dramatic drops in percent daily incidence since PAUSE. This is important, since it suggests a potential coupling between the strength of the population response to social distancing during an epidemic wave, and the subsequent epidemic outcome. Our results suggest that, when searching for indicators of this type of relationship, one may have to look within an extended window of over 4 weeks, in order to be able to capture consistent and measurable results, as further discussed in our last section.

The presence of positive correlations is replicated by the analysis in Appendix A in S1 Appendix, when using epidemic measures normalized by the number of tests performed. In Fig 10 in S1 Appendix, however, the initial transient windows are shorter, which may be explained by the fact that the initial negative correlations at PAUSE (as shown in Tables 5 and 6 in S1 Appendix) are weaker when dividing by the number of tests than the original ones (in Tables 1 and 2). This may be an effect of the fact that the availability of tests may have been higher in the very first stages of the epidemic in counties with higher infection rates, thus damping out the between-county differences in epidemic size.

### Correlation between traffic comeback and epidemic recovery after onset of PAUSE

As Figs 4 and 6 suggest, both traffic and mobility recovered from PAUSE at different rates and to different extents across New York State counties. As mentioned before, it is likely that the increase in mobility has much to do with the density and location, but also with the current epidemic reports in each county. Our last goal in this study is to investigate to what extent traffic/mobility comeback was correlated with the counties’ epidemic recovery in the 90 days after the start of PAUSE. We were able to do so for the Apple traffic (overall driving) data. Unfortunately, the frequent gaps in Google mobility data did not permit this longitudinal analysis for the mobility (location-specific) time series.

Similarly with the epidemic recovery measures, we computed, as a measure of traffic comeback by the *k*-th day after the start of PAUSE, the difference between the traffic level *A*(*k*) (as % of the baseline, on day *k* ≥ 10 after the start of PAUSE), and the traffic lowest level *A*_min_ (shortly following the start of PAUSE). Recall that all counties reached their lowest traffic value by *k* = 9 (with the few outliers exhibiting their minima a little before or after this window having maintained very close to minimum values within the window). Hence, for values of *k* ≥ 10, *A*(*k*) − *A*_min_ can be interpreted as a measure of traffic progression after it had reached its minimum.

**Fig 9:**
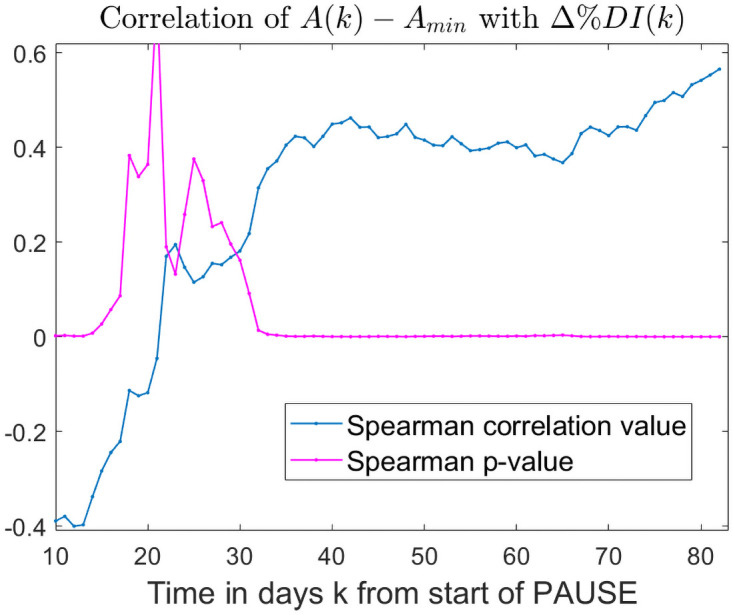
Correlations between traffic comeback and epidemic recovery after the start of PAUSE. *For each day k* ≥ 10 *after the start of PAUSE (represented along the horizontal axis), we computed the correlation between the comeback in driving from the lowest level after PAUSE A*(*k*) − *A*_min_ and Δ%*DI*. *Both measures were calculated for the 7-day window smoothed respective time series. The Spearman correlation values are shown in blue, and the p-values in purple*.

For each county, we calculated the Spearman correlation between the traffic comeback measure *A*(*k*) − *A*_min_, and the epidemic recovery measure Δ%*DI*, for all 10 ≤ *k* ≤ 83. Fig 9 shows the correlation values and the significance value for each day *k* after the start of PAUSE (represented along the horizontal axis). We observed a short window of 5 days where the two measures showed a significant negative correlation, followed by a transient window in which we found no significant correlations. Beginning about *k* = 30 days after the start of PAUSE, the two measures started to exhibit a consistent and significant *positive* correlation, which persisted until the end of the observed period (*k* = 83). In other words, when looking at patterns over 4 weeks from the start of PAUSE, lower comeback in driving based activities correlated with a lower (“more negative”) percent daily incidence, hence with better epidemic outcome.

## Discussion

Our study investigated epidemic and social mobility patterns in New York State counties between the advent of COVID-19 (March 2020), and until the first state-wide wave subsided (June 2020). We first focused on identifying the signature of the time series in each data set separately, and on drawing qualitative and quantitative comparisons between the situation in each county. We then aimed to understand the dynamic interplay between the two, by testing for correlations between measures describing size and progression of the epidemic and measures of traffic and population mobility to various targets (during PAUSE, as well as along the process of relaxing the PAUSE directives).

We found cumulative and daily incidence in each county at the start of PAUSE (both when used as direct measures and when reported as a population percent, or per number of tests performed) to show significant negative correlations with the lowest traffic/mobility level in each county (which followed promptly within a nine day window from the start of PAUSE). In other words, the higher the infection spread in a county (reflected in higher cumulative and daily incidence), the more drastic the subsequent drop in traffic/mobility (hence the lower the minimum level reached). Our results support the idea that psychology played a significant role in the shutdown process, and that counties with reportedly higher infection risk were able to enforce a more thorough lockdown protocol. However, we should keep in mind that significant correlations do not indubitably imply a cause-effect relationship, and that other potential explanations that may underlie or contribute to the observed correlations. For example, the higher return of international travelers in urban counties may have contributted to their larger initial outbreaks, and the mobility reduction in these counties may have been facilitated by the prevalence of high-income workers who could work remotely.

We also found a significant positive correlation between the lowest traffic/mobility levels following the start of PAUSE, and the change in epidemic daily incidence between the start of PAUSE and the end of the study (90 days later). In other words, the bigger the drop in traffic/mobility, the larger the drop in daily incidence, hence the better epidemic outcome. This supports the idea that PAUSE may have had a significant part to play in the process of curbing the infection rate, but also suggests a secondary mechanism: counties with high infection rates were more motivated to act more drastically and faithfully along the PAUSE directives, which lead to deeper cuts in infection. We noticed that this correlation emerges in fact a lot earlier than 90 days. The analysis of all traffic/mobility modalities in conjunction with the epidemic trends suggest that the correlation between the size of traffic/mobility drop and the epidemic recovery became significant around 4 weeks after the start of PAUSE. This is longer than the 14 day period that was a popular expectation with the general public (used in establishing individual quarantines).

Finally, we found a significant correlation between traffic comeback in each county from their lowest levels after the start of PAUSE, and the reduction in epidemic rate, in the sense that a more pronounced return to normal activities was associated with a less dramatic slowing down of the daily infection. As before, this correlation did not manifest immediately after the start of PAUSE, but rather became significant after a period of about 4 weeks.

Altogether, our findings support a potential tight and bidirectional dynamic coupling between the behavioral response of the population to mandated social distancing measures and the timeline of epidemic rise and recovery. While work beyond the limitations of correlation analyses would be needed to understand in more detail the underpinnings and consequences of this coupling, our study advocates for the potential importance of the PAUSE directive, of the degree and length to which it was observed. It also suggests that one may need to look for markers of the dynamic coupling between the epidemic and the population social response past the originally anticipated time frame from implementing the social measures.

## Supporting information

S1 Appendix (PDF)
