# Supplementary material for: Dynamic coupling between the COVID-19 epidemic timeline and the behavioral response to PAUSE in New York State counties
Source: PLoS One. 2021 Aug 4;16(8):e0255236. doi: 10.1371/journal.pone.0255236 (PMC8336843; doi:10.1371/journal.pone.0255236)
Supplement: S1 Appendix — (PDF) [file pone.0255236.s001.pdf]

S1 Appendix: Results of the correlation analyses, with testing normalization included

The Appendix reproduces the analysis in the main text, for epidemic measures normalized by the number of COVID-19 tests performed. In other words, the epidemic measures used here are: CI/T (cumulative incidence per total number of tests performed); DI/DT (daily incidence per daily number of tests performed). With these measures, the results in Tables 5 and 6 correspond to those in Tables 1 and 2 in the main text.

In addition, for each day $k \geq 10$ after the onset of PAUSE, we computed measures of epidemic recovery (similar to those used in the main text, but in this case including the normalization by the number of tests): $CI/CT(k) - CI/CT(1)$, the change in cumulative incidence per number of tests, between its value on the current day k, and its value at the start of PAUSE; $DI/DT(k) - DI/DT(1)$, the change in daily incidence per number of daily tests between its current value on day k and its value at the start of PAUSE (with the ratio set to zero for the days when no tests were performed). These were computed both for the original time series, and for the seven day moving window average time series for DI and DT; only results for the latter are illustrated (those for raw time series are similar). With these measures, Tables 7 and 8 correspond to those in Tables 3 and 4 in the main text, and Figures 10 and 11 correspond to Figures 8 and 9 in the main text.

	CI/T		DI/DT	
	Corr coef	p-value	Corr coef	p-value
**Traffic min**	-0.47	$< 10^{-3}$	-0.54	$< 10^{-5}$
**Retail min**	-0.51	$< 10^{-4}$	-0.47	$< 10^{-3}$
**Grocery min**	-0.31	0.01	-0.38	$< 10^{-2}$
**Work min**	-0.71	$< 10^{-9}$	-0.64	-10^{-7}

Table 5. Spearman rank correlations between epidemic incidence (normalized by testing levels) and our measure social mobility in New York counties at the time of PAUSE. *The epidemic measures (computed on the day PAUSE started) are: cumulative incidence per number of tests in each county (CI/T, columns 1 and 2); daily incidence per daily number of tests (DI/DT, columns 3 and 4). The first row shows the correlations of these epidemic measures with the lowest traffic level (which occurred briefly after the start of PAUSE), as a fraction of the original traffic baseline. The other three rows show the corresponding correlations with the lowest mobility to Retail, Grocery and Workspace (as a fraction of the baseline). The corresponding significance values are shown as separate columns.*

	Tuesday			
	CI/T		DI/DT	
	Corr coef	p-value	Corr coef	p-value
**Retail min**	-0.59	$< 10^{-6}$	-0.52	$< 10^{-4}$
**Grocery min**	-0.45	$< 10^{-3}$	-0.43	$< 10^{-3}$
**Work min**	-0.70	$< 10^{-9}$	-0.68	$< 10^{-8}$
	**Sunday**			
	CI/T		DI/DT	
	Corr coef	p-value	Corr coef	p-value
**Retail min**	-0.34	$< 10^{-2}$	-0.29	0.01
**Grocery min**	–	–	–	–
**Work min**	-0.57	$< 10^{-5}$	-0.54	$< 10^{-5}$

Table 6. Spearman correlations between measures of epidemic epidemic incidence (normalized by testing levels) and weekly measures of social mobility in New York counties at the time of PAUSE. *The epidemic measures are: cumulative incidence per number of tests in each county (CI/T, columns 1 and 2); daily incidence per number of daily tests (DI/DT, columns 3 and 4). The lowest mobility levels to Retail, Grocery and Workspace were computed separately for each day of the week; the table shows the results for correlations with Tuesday and Sunday time series.*

	**Change in CI/T** 90 days after start of PAUSE		**Change in DI/DT** 83 days after start of PAUSE	
	Corr coef	p-value	Corr coef	p-value
**Traffic min**	0.41	$< 10^{-3}$	0.55	$< 10^{-5}$
**Retail min**	0.48	$< 10^{-4}$	0.48	$< 10^{-3}$
**Grocery min**	0.34	0.0018	0.38	$< 10^{-2}$
**Work min**	0.59	$< 10^{-6}$	0.64	$< 10^{-7}$

Table 7. Spearman correlations between lowest traffic/mobility levels in New York counties shorty following PAUSE, and epidemic control by June 20, 2020. *As measures of epidemic control, we used the change in CI/T and DI/DT, between the start of PAUSE and the end of our study (June 20, 90 days after the start of PAUSE). These were correlated with the lowest traffic level, as fraction of traffic baseline (top row), and with the lowest mobility levels to Retail, Grocery and Workspace (remaining three rows). The corresponding significance values are shown as separate columns.*

	Tuesday			
	**Change in CI/T** 90 days after start of PAUSE		**Change in DI/DT** 90 days after start of PAUSE	
	Corr coef	p-value	Corr coef	p-value
**Retail min**	0.59	$< 10^{-6}$	0.53	$< 10^{-5}$
**Grocery min**	0.54	$< 10^{-5}$	0.44	$< 10^{-3}$
**Work min**	0.59	$< 10^{-6}$	0.68	$< 10^{-8}$
	**Sunday**			
	**Change in CI/T** 90 days after start of PAUSE		**Change in DI/DT** 90 days after start of PAUSE	
	Corr coef	p-value	Corr coef	p-value
**Retail min**	0.31	0.01	0.29	0.01
**Grocery min**	–	–	–	–
**Work min**	0.46	$< 10^{-3}$	0.53	$< 10^{-5}$

Table 8. Spearman correlations between lowest weekly mobility levels in New York counties shorty following PAUSE, and epidemic control by June 20, 2020. *As measures of epidemic control, we used the change in CI/T and DI/DT, between the start of PAUSE and the end of our study (June 20). The lowest mobility levels to Retail, Grocery and Workspace were computed separately for each day of the week; the table shows the results for correlations with Tuesday and Sunday time series.*

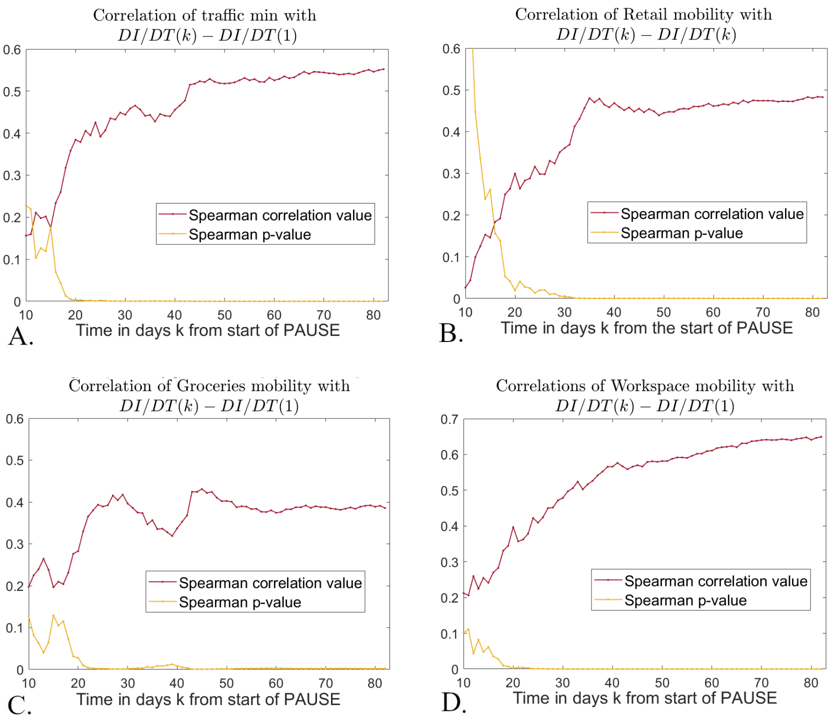

Fig 10. Spearman correlations between traffic/mobility lowest levels after PAUSE, and epidemic recovery before June 20, 2020. *For each day $k \geq 10$ after the start of PAUSE (represented along the horizontal axis) we computed correlations between the traffic/mobility lowest level and the epidemic recovery measure $DI/DT(k) - DI/DT(1)$. The values of $DI/DT(k) - DI/DT(1)$ were computed from the smoothed epidemic time series, for $10 \leq k \leq 83$ days. The correlation values are shown in brown, and the p-values in orange. Each row illustrates the results for a different traffic /mobility data set:* **A.** *driving, as percent of baseline;* **B.** *mobility to Retail,* **C.** *mobility to Grocery,* **D.** *mobility to Workspace, as percents of the reference day.*

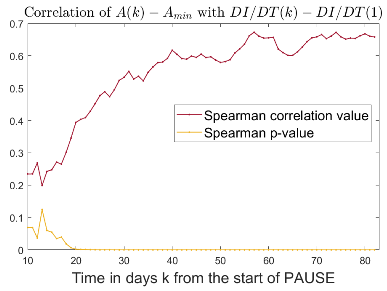

Fig 11. Correlations between traffic comeback and epidemic recovery after the start of PAUSE. *For each day $k \geq 10$ after the start of PAUSE (represented along the horizontal axis), we computed the correlation between the comeback in driving from the lowest level after PAUSE $A(k) - A_{min}$ and the change in DI/DT since its value at the start of PAUSE: $DI/DT(k) - DI/DT(1)$. For consistency, both measures were calculated for the seven day window smoothed respective time series. The Spearman correlation values are shown in brown for each day, and the significance p-values are shown in orange.*
